# Retrospective Analysis of the Effectiveness and Reversibility of Long-Acting Contraception Etonogestrel (Implanon^®^) in Common Marmosets (*Callithrix jacchus*)

**DOI:** 10.3390/ani11040963

**Published:** 2021-03-30

**Authors:** Sandra Roubos, Annet L. Louwerse, Jan A. M. Langermans, Jaco Bakker

**Affiliations:** 1 Animal Science Department, Biomedical Primate Research Centre, Lange Kleiweg 161, 2288GJ Rijswijk, The Netherlands; roubos@bprc.nl (S.R.); louwerse@bprc.nl (A.L.L.); langermans@bprc.nl (J.A.M.L.); 2 Department Population Health Sciences, Animals in Science and Society, Faculty of Veterinary Medicine, Utrecht University, 3584 CM Utrecht, The Netherlands

**Keywords:** common marmoset, contraception, etonogestrel

## Abstract

**Simple Summary:**

Due to the breeding success of common marmosets in captivity, colony managers need to achieve a balance between maintaining sustainable population numbers while preventing the breeding of surplus animals. Population control can be achieved by various methods, reversible and nonreversible. Long-acting reversible contraceptives are preferred, as they are not permanent and eliminate the logistical problems associated with the daily or weekly administration of oral or injectable contraceptives. Implanon^®^ (etonogestrel) is a widely used progestin-based contraceptive in marmosets with the theoretical advantages of being reversible and long-acting. However, no dose and efficacy data are available yet. In this study, we examined, by using electronic health records, the relationship between the use of one-fourth or one-third of an etonogestrel implant in female marmosets and the number of parturitions, interbirth interval, litter size, body weight, stillbirths and unintended pregnancies. We have concluded that etonogestrel implants are efficacious and safe to use in marmosets. Our data result in recommendations about the use of etonogestrel implants in marmosets. Our data can probably be extrapolated to other callitrichids.

**Abstract:**

Contraception is an important population control method for the colony management of primates housed in captivity. Etonogestrel (ENG) implants (i.e., Implanon^®^) are a widely used progestin-based contraceptive in common marmosets (*Callithrix jacchus*) with the theoretical advantages of being reversible and long-acting. However, no dose and efficacy data are available yet. Therefore, data from 52 adult female marmosets contracepted with ENG (one-fourth or one-third of an implant) housed at the Biomedical Primate Research Centre (BPRC, Rijswijk, The Netherlands) over the past 18 years were analyzed. Using an electronic database, a retrospective longitudinal cohort study was conducted to calculate the reproductive data before, during and after ENG use. The data show an effectiveness in preventing pregnancy of 99%. The implant was effective within one week after insertion. Unintended pregnancies did occur, but in 60% of these cases, the animals were already pregnant at the time of implant insertion. In these cases, healthy offspring were born despite the use of the implant. No stillbirths, neonatal deaths or maternal deaths could be linked to ENG use. After implant removal, 83% of the animals delivered healthy offspring. No difference in contraception efficacy was observed between the use of one-fourth or one-third of an implant. ENG achieved a contraceptive protection exceeding 99% and was shown to be reversible concerning fertility. To our knowledge, this is the first detailed analysis on the use of ENG in marmosets.

## 1. Introduction

The common marmoset (*Callithrix jacchus)* (hereafter, “marmosets”) is a New World monkey whose natural habitat is the Atlantic coastal rainforests of Northeastern Brazil. Nowadays, they are found throughout the world in almost every zoo and are also frequently used in biomedical research [1,2]. Marmosets adapt well to captive conditions, and self-sustaining breeding colonies can be established relatively easily. Marmosets have the highest potential fecundity of any anthropoid primate [3,4,5]: marmosets usually give birth twice a year (mostly twins and triplets), show no seasonal limitations, have a relatively short gestation period of approximately 144 days [6], have a fertile postpartum ovulation and reach sexual maturity at about 18 months of age [7]. Due to their breeding success, colony managers need to achieve a balance between maintaining sustainable population numbers while preventing the breeding of surplus animals.

Population control can be achieved by various methods. Prior to the introduction of contraception, the used methods included the housing of sexes separately, transfer of animals to other institutions and zoos and euthanasia of surplus animals. Surgical contraception techniques, like castration and vasectomy [8], became available but have the disadvantage of being nonreversible. Nowadays, reversible contraceptives are available, which are the perfect tool for applying balanced population control. Hormonal substitution methods such as birth control pills and implants are commonly used in exotic species housed in zoos (EAZA Group on the Zoo Animal Contraception website, 2021; https://www.egzac.org/; accessed on 1 March 2021). Long-acting contraceptives are preferred, as they eliminate the logistical problems associated with the daily or weekly administration of oral or injectable contraceptives. In humans, etonogestrel (ENG) implants (i.e., Implanon^®^ and Nexplanon^®^) are long-acting, with great efficacy [9,10,11,12]. ENG is released gradually into the female system. It effectively suppresses ovulation and prevents spermatozoa passage by alteration of the cervical mucosa. 

Providing effective contraception for marmosets is challenging because contraceptives are not specifically designed for use in marmosets. Moreover, only limited information is available about its use in callitrichids [13,14] (https://www.egzac.org; accessed on 1 March 2021). The extrapolation of doses from other species is suboptimal, as significant differences can exist between species in drug metabolism and plasma clearance. Moreover, detailed information about the effectiveness, reversibility and side effects of ENG in nonhuman primates (NHP) are lacking. Hypothetically, the effectiveness of the ENG implant could be diminished if the rate-releasing mechanism is disrupted by cutting the implant into one-fourth to one-third parts. In an effort to fill this gap of scientifically reported knowledge, a retrospective data analysis was performed, including effectiveness, reversibility and the side effects of a one-fourth to one-third part of subcutaneously (SC) inserted ENG implants in common marmosets housed in captivity. 

## 2. Materials and Methods

The animals received contraception for colony management or welfare reasons. No animals received ENG solely for the purpose of this study. All data were obtained in retrospect from the electronic health record database of animals that were housed at the Biomedical Primate Research Centre (BPRC, Rijswijk, The Netherlands). Ethical approval was not required for this study. All animals were housed in accordance with Dutch law and international ethical and scientific standards and guidelines (EU Directive 63/2010). All husbandry procedures were compliant with the above standards and legislation. The animal care at BPRC is in accordance with the programs accredited by the Association for Assessment and Accreditation of Laboratory Animal Care International (AAALAC International). 

### 2.1. Animals

The demographic and reproductive data used in this study were obtained from the electronic health record database of the marmoset breeding colony at the BPRC. This marmoset colony was founded in 1975 and consisted initially of marmosets obtained from various accredited suppliers (only captive-bred marmosets were included). Later, new breeding lines were introduced on several occasions to maintain the outbred characteristic of the colony. The colony continuously included around 15 breeding groups comprising a total of approximately 150 marmosets, ranging from infants to adults older than 12 years [15].

The breeding groups were housed in enclosures with access to an outdoor compartment as monogamous breeding pairs with successive sets of offspring [16]. The offspring remained with their family group for as long as possible—that is, until either the dam or sire or both parents rejected them or until they were selected for experimental use (>1.5 years old). Animals were in visual and vocal contact with at least one other group. Standard enrichment materials, such as branches, baskets, garden hoses, fire hoses and nets, were always present in the enclosures 16]. The temperature of the inside compartment was maintained between 22 and 25 °C, with a relative humidity between 50% and 60% and with a 12:12-h light:dark cycle (lights on, 0700 a.m.–0700 p.m.). Lighting was provided using full-spectrum fluorescent bulbs placed close to the cages, in addition to natural light through the windows. The room ventilation rate was around eight air changes hourly. The daily diet consisted of commercial primate pellets for New World Monkeys (Sniff, Soest, Germany) offered ad libitum and supplemented with limited amounts of fresh fruit, vegetables, Arabic gum and homemade porridge. Tap water was provided ad libitum in drinking bottles or via an automated drinking system. Details of animal management, husbandry and enclosures at the BPRC were described by Bakker et al. 2018. Animals were observed twice daily by a caretaker for (ab)normal (sexual) behavior.

As part of routine husbandry, body weights of all marmosets were obtained, on average, once-weekly. Weights were taken using a balcony box or placing a scale in the animal’s home cage by using positive reinforcement training (PRT).

The dataset used for the analysis covered the period from August 2002 until October 2020. The dataset consisted of a total of 52 captive-born female common marmosets (*Callithrix jacchus*) and included data on body weight, age, date of parturitions, litter size, still births and abortions, date of implant insertion and removal and the results of pathology (only if an animal needed to be euthanized).

### 2.2. Implant

The effectiveness and reversibility of ENG implants, available in Europe as Implanon^®^ (N.V. Organon International, Oss, The Netherlands), containing 68-mg ENG was analyzed.

**Figure 1:**
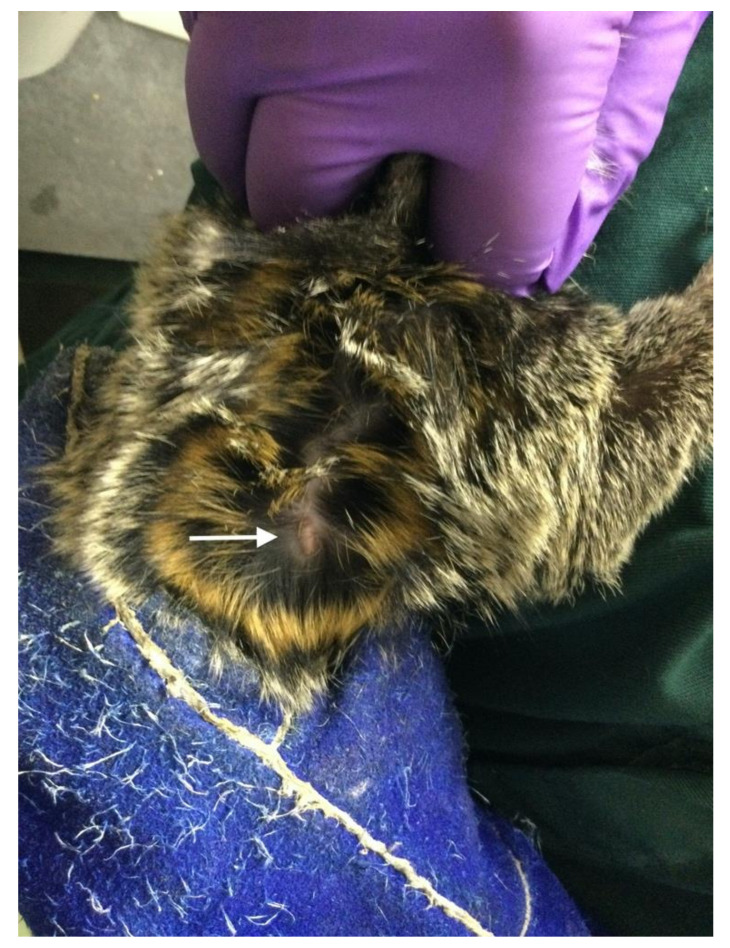
Photograph of a subcutaneous inserted etonogestrel (ENG) implant between the scapulae in a marmoset (arrow points to the implant position). Shaving is not necessary for inserting and for visual confirmation of the presence of the implant. When inserted correctly, the implant is clearly visible through the skin. The viewer’s orientation is the head of the animal at the top of the photograph.

In the current study, hormone values were not determined to monitor the ovarian cycle stage before, during or after implant insertion. For implant insertion, animals were not sedated, as with PRT training, the animals are trained to cooperate with capture and restraint to minimize the stress of this routine procedure. Abdominal palpation of the uterus was performed and appeared to be negative for pregnancy in all 52 animals at the moment of implant insertion. The implant was cut into 3 to 4 parts with a scalpel blade. The remaining parts were stored for a maximum of 6 months in a refrigerator (3–5 °C) until usage in other animals. The dosage was not dependent on the weight of the animal. The two used dosages were one-fourth or one-third parts. The implant was inserted with a manually loaded disposable applicator SC, into the tissue layer between the skin and the muscle, between the scapulae (Figure 1). The insertion site was not shaved nor disinfected. The insertion site did not require sutures. Immediately after the procedure, animals were allowed to return to their partner or family. After 3 years, the implant was removed and replaced by a new one if further contraception was required.

### 2.3. Pathology

The animals included in this study that were euthanized (for various reasons) were examined by a veterinary pathologist, with special attention to the reproductive tract.

### 2.4. Study Methods and Data Analysis

We conducted a retrospective longitudinal cohort study using electronic health records to calculate the reproductive data of 52 animals. All statistical analyses were performed with the R language and environment for statistical computing version 3.5.1 (R Foundation for Statistical Computing, Vienna, Austria. ISBN 3-900051-07-0, URL: http://www.R-project.org; accessed on 1 March 2021). A value of *p* < 0.05 was considered significant. Data are presented as mean ± standard deviation. Normality was tested with the Shapiro–Wilk test. 

Age, parturition, interbirth interval, body weight and number of offspring per parturition were determined before, during and after ENG implantation. Reproductive output per litter per parturition was defined as the number of infants produced at full-term gestation, dead and alive. The gestation period of the common marmoset was set at 144 days [6]. As the ENG dose was not described to affect the gestation and/or gestation period, estimated date of conception was calculated by subtracting 144 days from the parturition date. The restoration of reproductive potential following the removal of the implant was calculated as well. Contraceptive efficacy and failure rate of the ENG implant were calculated by the number of unintended pregnancies divided by the potential number of ovarian cycles of all implanted nonpregnant animals [12,17]. One ovarian cycle was set at 28 days [18].

It is described that the estimation of the diagnosis of pregnancy by abdominal palpation is the most reliable after 41 days [19]. Animals carrying the implant less than 56 consecutive days were excluded from the data analysis for the effectivity assessment of ENG, as the start of efficacy was described to be 14 days (https://www.egzac.org; accessed on 1 March 2021).

The body weight change during ENG use was calculated by comparing the body weight at the moment of ENG implant insertion with the body weight one year later. For this reason, animals with ENG less than one year were excluded from this body weight analysis. Additionally, animals that became pregnant during this year were excluded from the analysis, as pregnancy influences the body weight of the animal. A two-sided paired *t*-test was performed to calculate body weight changes. 

Dose dependency was tested with Fisher’s exact test (sample was too small for the chi-square test). The difference in the number of offspring (normally distributed) was tested with paired *t*-tests.

Marmosets are described not to menstruate, nor do they show other external indications of the ovarian cycle [19,20]. Therefore, the only moment the cycle stage can be reliably determined is the postpartum period. The first ovulation generally occurs within 10 to 20 days postpartum [1,5,6,21].

## 3. Results

### 3.1. Data Pre-ENG 

The mean ages of the animals at the time of ENG insertion were 5.42 ± 1.91 years. The mean body weights at the time of implant insertion were 343 ± 65 g. From the 52 animals, 46 were multiparous, and six were nulliparous. All included animals had a total of 290 parturitions before the insertion of ENG. Two suspected abortions were noted in the respective patient files of the animals. The data are shown in Table 1.

### 3.2. Start of Contraceptive Efficacy of ENG after Administration

To determine the start of the contraceptive efficacy of ENG, the data of the animals that received ENG < 20 days postpartum were analyzed. Twenty-five animals met the inclusion criteria. ENG was inserted at a median of 9.5 days (0–202 days) postpartum. In 23 animals, the implant was inserted within four days postpartum. None of these animals conceived. No animals were implanted between 5–14 days postpartum. Two animals received ENG 15 to 16 days postpartum, and those two animals both conceived. No animals were implanted 17–20 days postpartum.

### 3.3. Data during ENG

The results are shown in Table 2. Eleven animals implanted with ENG had 15 confirmed unintended pregnancies (confirmed by parturitions and at necropsy), which resulted in 13 parturitions. All parturitions were uneventful, and the offspring were healthy. In nine of the 11 animals (animal no. one–nine), the time between implant insertion and parturition was < 144 days. Abdominal palpations for pregnancy appeared to be negative at the moment of implant insertion. However, the data suggest that these animals were already pregnant at that moment. Two of the 11 animals were euthanized before parturition and were diagnosed pregnant at necropsy. Unfortunately, no estimation was made about how long those two animals were pregnant (Table 2, animal no. three and six). The 11 animals received their ENG 67.3 ± 43.4 days postpartum. These animals had a mean number of offspring of 2.17 ± 0.61, with 94% born alive. Before ENG, the mean number of offspring of these animals was 2.46 ± 0.50, with 92% born alive (Table 1). The difference in the number of offspring was not significant (*p* = 0.42).

Forty animals met the inclusion criteria to calculate the effectivity. There was a total time of being implanted of 25,308 days. Calculated with an ovarian cycle of 28 days, ENG covered 903.9 cycles for possible conceptions. Out of these 903.9 cycles, only six proven pregnancies occurred (failures of the ENG implant). For calculation of the failure rate, these six complete pregnancies were subtracted from the total time (6 × 144 days). Moreover, animals no. one–nine from Table 2 were pregnant during a part of this “implanted period”, so these 823 days were subtracted from the total time as well, resulting in a total of (25,308 − 1687)/28 = 843.6 cycles covered by ENG. Failure rate: 6/843.6 × 100 = 0.71%. With this result, the effectiveness was calculated: 100% − 0.71% = 99.3%.

### 3.4. Duration of Contraceptive Effects of ENG

Of the 52 animals, 25 animals had the implant for less than a year. Seventeen animals had the implant between one and two years. Ten animals had the implant more than two years, with six of these ten animals having the implant for more than three years.

The duration of the efficacy could not be calculated due to insufficient amounts of data.

### 3.5. Data Post ENG

In ten animals, the implant was removed. Subsequently, six of these animals were allowed to reproduce by housing them together with a fertile male. Two out of these six animals were euthanized, respectively, 93 (pregnant at necropsy) and 108 days after implant removal. The other four animals delivered healthy offspring. The details of those four successful breeders after implant removal are shown in Table 3. Conclusively, after implant removal, fertility was restored in five out of six animals (83%). However, the time between implant removal and the first parturition was, in three out of four animals, longer compared to the interbirth interval thereafter.

Pregnancies pre-, during and post-ENG showed no significant differences in the number of animals born alive per parturition (Table 4). The difference in the number of offspring was also not significant (respectively, *p* = 0.42 during and *p* = 0.42 after ENG). Out of the six included nulliparous animals, four never gave birth. Those four animals were housed with males, but as there was no desire to breed with these animals, ENG was inserted.

### 3.6. Second Implantation

Ten animals were implanted for a second time. Five of these animals were also described in “Data during ENG” (Table 2), four animals were also described in “Data post-ENG” (Table 3) and only one animal was not described in detail before. This animal’s implant had not yet failed, but during a routine physical examination of this animal, the implant could not be palpated at the back of the animal, so it received a new implant. This was 201 days after the first implant; it stayed in for 217 days. None of the ten animals with suggested implant failure of the first implant conceived after receiving a new implant (Table 2, animals no. 2, 5, 7, 10 and 11).

### 3.7. Pathology

The animals included in this study that were euthanized (for various reasons) were examined by a veterinary pathologist with special attention to the reproductive tract (*n* = 32). At necropsy, only one animal presented genital tract abnormalities described by the pathologist as endometriosis. This animal also presented general macroscopic and histologic lesions suggestive of Marmoset Wasting Syndrome.

### 3.8. Body Weight Change

Twenty animals met the inclusion criteria to calculate body weight changes during contraceptive use. At the moment of implant insertion, the mean body weight of these animals was 365 ± 54 g. After one year of implant carriage, the weight increased significantly to 390 ± 54 g (*p* = 0.01).

### 3.9. Dose

The used doses were one-fourth 28 times and one-third 19 times. In four animals, the dose was not registered, and in one animal, a one-half ENG implant was inserted. In the five animals with unintended pregnancies (Table 2, animals no. 2, 7, 9, 10 and 11), three animals received one-fourth and two animals one-third. No difference was found between doses in contraceptive efficacy and failure (*p* = 0.99).

## 4. Discussion

The present study shows the efficacy and reversibility of ENG as a contraceptive in the common marmoset. Although ENG is already used in callitrichids [13,14] (https://www.egzac.org; accessed on 1 March 2021), no literature about the efficacy and reversibility of ENG is available. ENG use in other NHP is mentioned but not further specified [13,22]. 

Our data show that ENG is a safe and reliable contraceptive for use in marmosets (99.3% efficacy). The efficacy rate in our analysis was probably underestimated, as our data showed that most of the unintended pregnancies were not the result of actual ENG failures but instead occurred because the implant was inadvertently not inserted or the animals conceived prior to the insertion or shortly after the insertion of the implant.

A problem common to all NHP is the risk of losing the implant due to active or accidental removal by the individual animal or its social companions—for example, during grooming. Unfortunately, there was no systematic check for confirmation of the presence of the implant in the animals included in this study. Implant loss was suggested in five animals, as none of these animals conceived after receiving a new implant (Table 2, animals no. 2, 5, 7, 10 and 11). In only two of these animals, the implant was palpated and subsequently removed while inserting a new implant. The records of the other three animals contained no information about an implant presence. A check for the presence of the implant can be supported with ultrasound and radiography in future studies and use. The implants contain barium sulphate, which makes the implants visible on radiographs [23]. Six nulliparous animals were included in the analysis. Since infertility is uncommon in marmosets, as they have the highest potential fecundity of any anthropoid primate [3,4,5], the expectation was that these animals would be fertile without the implant. These six nulliparous females did not conceive during ENG, confirming the efficacy of the implant. In humans, unintended pregnancies associated with ENG have also been described [12,24]. Our efficacy data in marmosets (99.3%) are comparable to that in humans (more than 99%). However, it cannot be excluded that the number of failures is underestimated in our study, as the resorption of fetuses could have been missed. The described cases of failure included pregnancy prior to implant insertion, incorrect timing of insertion, expulsion of the implant, interaction with hepatic enzyme-inducing medicines and product/method failures.

All parturitions of the ENG-implanted animals were uneventful. This supports the theory that progestins do not interfere with parturition. It has been described that many callitrichid females have been purposefully implanted while pregnant to prevent the fertile postpartum estrus [14]. The first ovulation generally occurs within 10 to 20 days after parturition [1,5,6,21]. McNeilly et al. [25] reported that the time from parturition to conception was approximately 28 days longer for animals that were lactating; however, the females of that study ovulated while lactating. Theoretically, it is contraindicated to use ENG during lactation, as the hormone gets excreted in the milk. As the current study focusses on female animals and pregnancies, conclusions about lactation are beyond the scope of this investigation.

In humans, one complete intact implant has to be inserted to achieve full infertility. Since marmosets are smaller and weigh less, it is desirable not to use a full implant. A concern was that the effectiveness of the ENG implant as a contraceptive could be diminished if the rate-releasing mechanism was disrupted by cutting the implants into one-fourth to one-third parts. Implanon is described as a flexible rod composed of a solid core of ethylene vinyl acetate (EVA) with crystals of ENG imbedded within the core. Surrounding the core is a thin layer of EVA (0.06-mm thick) controlling the released ENG rate. The in vitro data show that, when an implant is broken or bent, the release rate of ENG is slightly increased. It is unknown if an implant remains as effective after a fracture in vivo. In humans, the fracture of an implant in situ is rare, and little is known about the clinical consequences [26,27,28,29,30]. There is no official recommendation by the manufacturers on when to remove a fractured ENG implant. Our data, in which all ENG implants were cut into parts, showed that the ENG implants remained effective after cutting. As two-thirds up to three-fourths of the cut implant parts were stored before use and the contraceptive efficacy was 99.3%, this suggests that the storage of the cut parts in the refrigerator for up to six months did not appear to affect the efficacy.

In humans, threshold concentrations of the hormone were described to be reached in the blood within eight hours following SC insertion if inserted during the first five days of the ovarian cycle, which starts with the first day of bleeding. The implant is effective after seven days if it is inserted at any other time in the ovarian cycle [31,32]. In our study, 23 animals received the ENG implant within four days postpartum. None of these animals conceived. This shows that the ENG implants are effective within one week, as the first postpartum ovulation generally occurs 10 to 20 days postpartum. These data are comparable to women, where it is described to be immediately effective if inserted during the first five days of their ovarian cycle, i.e., in our study, the first four days postpartum [9,10,11]. 

In human clinical trials, fertility is described to return after the removal of ENG [12]. The marmosets’ fertility was restored in five out of six animals after removal of the ENG implant. However, the sixth animal had to be euthanized unfortunately 108 days after implant removal. At necropsy, this animal showed no pregnancy. In our study, the time between implant removal and first parturition was in three out of four animals, longer compared to their interbirth interval thereafter, all suggesting a delayed restoration of the fertility post-ENG. Further research is needed into the reversibility of the fertility in marmosets.

A possible confounding factor is the increase of the female ages during the time the contraceptive was used. The effect of maternal age on reproduction in marmosets is not fully clear. Reproductive senescence was not described by Abott et al. [1] and Box and Hubrecht [33]. Although we only have data for four reproducing animals post-ENG, our data are in agreement with those reports, as the difference in the number of offspring pre- and post-ENG use was not significant in our study. In contrast, some age effects on litter size and interbirth interval have been described, but these were small [4]. The indirect factors possibly influencing the reproductive variation, such as group size (i.e., the number of potential helpers) and group composition, were not analyzed. Future analyses should include all these parameters.

The described side effects of ENG in humans are possible weight gain and the increased or decreased frequency of bleeding during menstruation. In humans over a two-year period, ENG users had an average increase of 2.6% of their initial body weight [34]. However, this average weight increase was documented even among users of nonhormonal methods of contraception, which suggests that weight changes over time are influenced by a complex interplay of multiple factors [35]. The current study in marmosets showed a significant body weight increase over a one-year period. However, the indirect factors possibly influencing the body weight developments, such as aging, diet changes and group sizes (i.e., number of potential helpers), were not analyzed. Future analyses should include all these parameters.

One study investigated the effect of ENG on the behavior of Barbary macaques, showing that ENG can result in increased aggression, more self-scratching and more time self-grooming, demonstrating anxiety [22]. In our colony, the animals were observed twice-daily by a caretaker for (ab)normal (sexual) behavior. Although not studied in full detail, no abnormal behavior related to ENG was noted.

In contrast to humans, marmosets are described not to menstruate, nor do they show other external indications of the sexual cycle [19,20], and therefore, the absence of menstruation is not indicative of the efficacy of ENG in marmosets. The detection of ovulation is best achieved by endocrine monitoring using hormone assays. Although noninvasive methods for the routine monitoring of the reproductive state and reproduction control in large colonies of captive marmosets have been developed [21], they were not applied at the BPRC, as there was no clinical need. Moreover, under laboratory conditions, marmosets are described to mate throughout the ovarian cycle and are not restricted to the periovulatory period [36]. Therefore, sexual behavior is no marker of the ovulation status in marmosets.

In total, 33 necropsies were performed in animals that received ENG at some point during their life. One case of endometriosis was found at necropsy. Spontaneous endometriosis is described to occur only in women and menstruating Old World monkeys [37] but was not described in marmosets. However, our animals were also found with lesions suggestive of Marmoset Wasting Syndrome. Therefore, a link between alterations of the genital tract and the use of ENG is not suspected. This is in line with the human data [38]. 

The human dose is 68mg ENG (= one whole implant) for a typical human female of about 70 kg (= approximately 1 mg/kg). At the BPRC, one-fourth to one-third parts of the implants were inserted SC between the scapulae. By using one-fourth to one-third parts in marmosets weighing 400 g, the doses were 42.5–56.7 mg/kg. However, an important consideration for all callitrichids is that they probably require a much higher dose of the steroid hormone-based contraceptives to achieve downregulation, probably because they have higher endogenous levels of steroids, likely resulting in a decreased binding affinity of the hormone receptors [39]. The generally accepted estimates are 10–20× the Old World monkeys’ dose, which would include the human dose (personal communication, Sally Boutelle, AZA Wildlife Contraception Center, St. Louis, MO, USA). However, in the current study, 28 animals received one-fourth parts and 19 animals one-third parts. No difference was observed between the dose and failure rate/efficacy. We can conclude that the one-fourth to one-third parts do not affect the contraception efficacy in marmosets, but this might be due to overdosing. If this is the case, no side effects from overdosing were observed. According to the EAZA guidelines, it is advised to change the implant after two to three years, although there is a human study showing that ENG in humans is effective for four to five years instead of three years [40]. In this study, we did not obtain data to provide a conclusion about the duration of contraception with the implant over a period longer than three years. 

The preferred implant location in humans is the inner aspect of the nondominant arm. However, in marmosets, it is preferred to insert the implant on the back of the animal, between the shoulder blades, to minimize interference with the implant by the animal and subsequent trauma. In our study, the animals showed no aversive reactions to the implant, e.g., by scratching the implant site. The implant location was not expected to influence the efficacy.

Our data could be biased by social cues and conditioning, as those factors play an important role in reproductive behaviors and controlling reproductive cycles in marmosets. Socially subordinate female marmosets show suppressed ovulation cycles and sexual behavior when around a dominant female [1,7,41,42]. The most common reason to use ENG was because the condition of the animals was slowly deteriorating, indicative of Marmoset Wasting Syndrome. Behavior and olfactory cues from the dominant female affect the establishment and maintenance of ovulatory suppression of subordinate female cage mates [7]. Hypothetically, the loss of the condition of the dam could create a loss of her dominant position, which could result in her daughter ovulating. Additionally, introducing ENG in the dam—thus, the dam becoming nonovulating—could result in the ovulation of her daughters. The detection of ovulation is best achieved by endocrine monitoring using hormone assays. Unfortunately, our study did not include the monitoring of ovulation by hormone assays of the dams and their daughters. Future analyses should include hormone assays and reproduction parameters of dams and their daughters.

In the present study, we examined the relationship between the use of ENG and the number of parturitions, interbirth interval, litter size, body weight, stillbirths and unintended pregnancies in marmosets (efficacy–failure rate of the ENG implants). We concluded that ENG implants are efficacious and safe to use in marmosets. Given the evidence presented here, we recommend the timing of implant insertion to be at the last stage of pregnancy and up to three days postpartum to prevent the fertile postpartum estrus. The dose of the implant can be a one-fourth or one-third part. It is advised to check for the presence of the implant routinely. 

## Figures and Tables

**Table 1:** The reproduction data of all 52 included animals. The values are presented as the mean ± standard deviation. The ovarian cycle was set at 28 days, and full-term pregnancy was set at 144 days. The failure rate is defined as the number of unintended pregnancies divided by the potential number of ovarian cycles of all implanted nonpregnant animals. ENG: etonogestrel.

	All Animals (*n* = 52)	Animals with ENG > 55 Days (*n* = 40)	Animals Giving Birth during ENG (*n* = 11)
Age at implant placement (years)	5.42 ± 1.91	5.31 ± 2.06	5.63 ± 2.24
Body Weight (grams)	343 ± 65	348 ± 62	332 ± 55
Number of parturitions	5.58 ± 3.75	5.50 ± 3.86	5.45 ± 3.62
Interbirth interval (days)	183.63 ± 25.05	181.89 ± 23.15	188.66 ± 26.55
Number of offspring per parturition	2.44 ± 0.48	2.41 ± 0.51	2.46 ± 0.50
Live offspring (%)	0.92 ± 0.17	0.91 ± 0.19	0.92 ± 0.15
Days of ENG use	25,554	25,308	-
Theoretical number of cycles during ENG use	912.6	903.9	-
Theoretical number of cycles during ENG use after correction for pregnancy	852.4	843.6	
Failure rate	0.70%	0.71%	-

**Table 2:** The data of 11 animals giving birth during ENG. In nine of the 11 animals (animals no. 1–9), the time between implant insertion and the first parturition was <144 days, suggesting that they were already pregnant at the moment of implant insertion. Animals no. 3 and 6 were euthanized for reasons not related to this study and were diagnosed pregnant at necropsy. In animals no. 2, 7 and 9, one or more parturitions took place after having ENG for more than 144 days, suggesting implant failure. Animals no. 2, 5, 7, 10 and 11 received a new implant. Animals that received a new implant did not conceive thereafter.

Animal No.	Days between Implant Insertion and First Parturition	Suggested Implant Failure	Subsequent Parturitions in Days after Implant Insertion	Suggested Implant Failure	New Implant Insertion
1	134	no		-	-
2	134	no	583 days after implant	Yes	Yes, 585 days after 1st implant insertion
3	123	no		-	-
4	142	no		-	-
5	54	no			Yes, 4.19 years after 1st implant insertion
6	85	no			-
7	121	no	277 and 430 days after implant insertion	Yes, twice	Yes, 436 days after 1st implant insertion
8	28	no			-
9	2	no	296 days after implant insertion	Yes	-
10	778	Yes		Yes	Yes, 783 days after 1st implant insertion
11	368	Yes		Yes	Yes, 341 days after first implant insertion

**Table 3:** Pregnancy post-ENG. In 10 animals, the implant was removed. Subsequently, six of these animals were allowed to reproduce. Four animals (A–D) gave birth and are presented below in detail. One hundred percent live offspring were born.

Animal	A	B	C	D
Age at moment of ENG insertion and ENG removal (years)	6.5 and 7.7	6.2 and 7.4	3.1 and 3.3	1.4 and 1.9
Body weight at moment of ENG insertion and ENG removal (g)	300 and 358	323 and 329	525 and 395	427 and 434
First parturition after implant removal (days)	154	281	234	381
Number of parturitions	2	1	8	2
Interbirth interval (days)	158	-	173	230
Number of offspring per parturition	3	2	2	3
Age at new implant (years)	8.6	8.2	7.3	4.3
Duration new implant (days)	687	717	480	16

**Table 4:** Number of offspring and number of animals born alive per parturition before, during and after ENG. Two animals that were implanted with ENG were pregnant during autopsy. They count in the number of pregnancies but not in the number of parturitions.

	Number of Offspring per Parturition	Proportion of Live Offspring (Mean ± Standard Deviation) per Parturition
Before ENG (*n* = 52 animals, 290 parturitions)	2.44 ± 0.48	0.92 ± 0.17
Pregnancies during ENG (*n* = 11 animals, 13 parturitions)	2.17 ± 0.61	0.94 ± 0.13
Pregnancies after ENG removal (*n* = 4 animals, 13 parturitions)	2.31 ± 0.63	1.00 ± 0

## Data Availability

No new data were created or analyzed in this study. Data sharing is not applicable to this article.
